# A Novel Acetone Sensor for Body Fluids

**DOI:** 10.3390/bios14010004

**Published:** 2023-12-22

**Authors:** Oscar Osorio Perez, Ngan Anh Nguyen, Asher Hendricks, Shaun Victor, Sabrina Jimena Mora, Nanxi Yu, Xiaojun Xian, Shaopeng Wang, Doina Kulick, Erica Forzani

**Affiliations:** 1 School of Engineering for Matter, Transport and Energy, Arizona State University, Tempe, AZ 85287, USA; oosoriop@asu.edu (O.O.P.); annguye6@asu.edu (N.A.N.); ajpete20@asu.edu (A.H.); 2 Center for Bioelectronics and Biosensors, Biodesign Institute, Arizona State University, 1001 S McAllister Ave., Tempe, AZ 85281, USA; svictor4@asu.edu (S.V.); smora2@asu.edu (S.J.M.); nanxiyu@asu.edu (N.Y.); xiaojun.xian@sdstate.edu (X.X.); shaopeng.wang@asu.edu (S.W.); 3 School of Molecular Sciences, Arizona State University, Tempe, AZ 85287, USA; 4 Department of Electrical Engineering and Computer Science, South Dakota State University, Brookings, SD 57007, USA; 5 School of Biological and Health Systems Engineering, Arizona State University, Tempe, AZ 85287, USA; 6 Mayo Clinic, Scottsdale, AZ 85289, USA; kulick.doina@mayo.edu

**Keywords:** wearable sensor, point of care, metabolic rate, fat oxidation, fat burning, ketones, breath sensor, digital medicine

## Abstract

Ketones are well-known biomarkers of fat oxidation produced in the liver as a result of lipolysis. These biomarkers include acetoacetic acid and β-hydroxybutyric acid in the blood/urine and acetone in our breath and skin. Monitoring ketone production in the body is essential for people who use caloric intake deficit to reduce body weight or use ketogenic diets for wellness or therapeutic treatments. Current methods to monitor ketones include urine dipsticks, capillary blood monitors, and breath analyzers. However, these existing methods have certain disadvantages that preclude them from being used more widely. In this work, we introduce a novel acetone sensor device that can detect acetone levels in breath and overcome the drawbacks of existing sensing approaches. The critical element of the device is a robust sensor with the capability to measure acetone using a complementary metal oxide semiconductor (CMOS) chip and convenient data analysis from a red, green, and blue deconvolution imaging approach. The acetone sensor device demonstrated sensitivity of detection in the micromolar-concentration range, selectivity for detection of acetone in breath, and a lifetime stability of at least one month. The sensor device utility was probed with real tests on breath samples using an established blood ketone reference method.

## 1. Introduction

The measurements of metabolic rate and acetone are of interest for weight management and overall health applications [1,2,3,4,5,6,7,8,9]. While the metabolic rate allows us to determine the rate of ATP molecules produced at a cellular level to sustain metabolic functions [10], ketones are biomarkers of fat oxidation. Along this line, ketone bodies are produced in our liver as a result of lipolysis [11]. This causes the release of β-hydroxybutyric acid and acetoacetic acid in the blood and the corresponding release of the decarboxylation byproduct, acetone, in our breath [12,13] and skin. Monitoring ketone production in the body is important for several reasons. Monitoring ketone levels is suggested for people who use caloric intake deficit methods to reduce body weight or use fat/protein rich and low carbohydrate diets to achieve a sustained state of higher blood ketone levels [14]. This state is known as ketosis. Diets rich in fat and low in carbohydrates have been shown to help children with epilepsy overcome seizures [15,16]. In addition, they have also shown positive results in connection with weight loss [17] and heart congestive failure [18]. On the other hand, in patients with type 1 diabetes, the absence of insulin can result in excessive accumulation of ketone bodies in the blood [19]. This state is known as ketoacidosis, which results in a lowering of blood pH [20,21]. Ketoacidosis is a potentially life-threatening condition for which patients need to constantly monitor their ketone levels [22,23]. 

Current methods to monitor the state of ketosis or ketoacidosis include urine dipsticks, electrochemical capillary blood monitors, and breath analyzers. Urine dipsticks are qualitative measurements that are influenced by the person’s hydration state [24,25]. Capillary blood measurement is more reliable for monitoring ketosis and currently is an approved method for use both at home and in clinical settings [26]. Though more reliable than urine, blood measurements can be invasive and painful. On the other hand, breath analysis is more acceptable because it is non-invasive and conveniently able to avoid disturbance to the patient [27]. The dominant ketone in breath is acetone due to the high volatility of the compound [7,8,28,29].

Breath acetone has been shown to be a reliable indicator of ketosis, and it correlates with the levels of blood ketones [30,31]. Most of the clinical studies showing these correlations are carried out using gas chromatography (GC) coupled to a chemical identification method [32,33,34], selected ion flow tube mass spectrometer (SIFT-MS) [7,8], or cavity-ring spectroscopy [35]. GC lacks real-time analysis capabilities, making it a difficult tool to use in a clinical setting, whereas SIFT-MS has been used for online real-time analysis of breath samples and potentially could be further implemented if the right equipment and technical assistance are available [36,37]. Several successful studies with accurate and real-time analysis of breath acetone levels using SIFT-MS have been reported [38,39,40]. More recently, cavity-ring spectroscopy has been used in a clinical study reporting the benefits of monitoring breath acetone over hydroxybutyrate for low carbohydrate diets [35].

Devices for the detection of breath have been described for over three decades already, with the first pioneering work cleared by the FDA in 1987–1988 [41,42] and publications by Kundu et al. [43,44]. Some breath acetone devices have been based on enzymatic reactions [45], semiconductor (metal oxide)-based sensors [46,47,48], colorimetric sensors [2,4,9,49], photoionization [48] and UV [50] detection. In addition, individual sensors for breath acetone have been developed using novel ideas [51,52,53,54]. More recently, commercial breath acetone sensors have been made available to the general public [48]. Most commercial breath acetone sensors (e.g., Ketonix and Biosense) are based on metal oxide semiconductor detectors (MOS sensors), as summarized by Alkdeh and Priefer [48]. In addition, several patents and patent applications have been published for acetone breath analysis [55,56,57,58,59,60,61]. Commercially existing systems need periodic calibration to perform accurately, and systems presented as intellectual properties have multiple reaction steps or rely on mechanically actuated parts.

Here, we present a novel acetone sensor device that aims to simplify existing thermally, mechanically, and multi-reactive assisted approaches. Furthermore, the system aims to relieve users from the calibration burden by incorporating a chemical pre-calibration process during sensor fabrication. The key element of the device is a robust sensor with the capability to measure acetone specifically, using a complementary metal oxide semiconductor (CMOS) chip and convenient data analysis from a red, green, and blue component deconvolution imaging approach. The utility of the sensor is also demonstrated with real tests on breath samples using reference methods established in the field.

## 2. Materials and Methods

### 2.1. Sensing Technique

**Figure 1:**
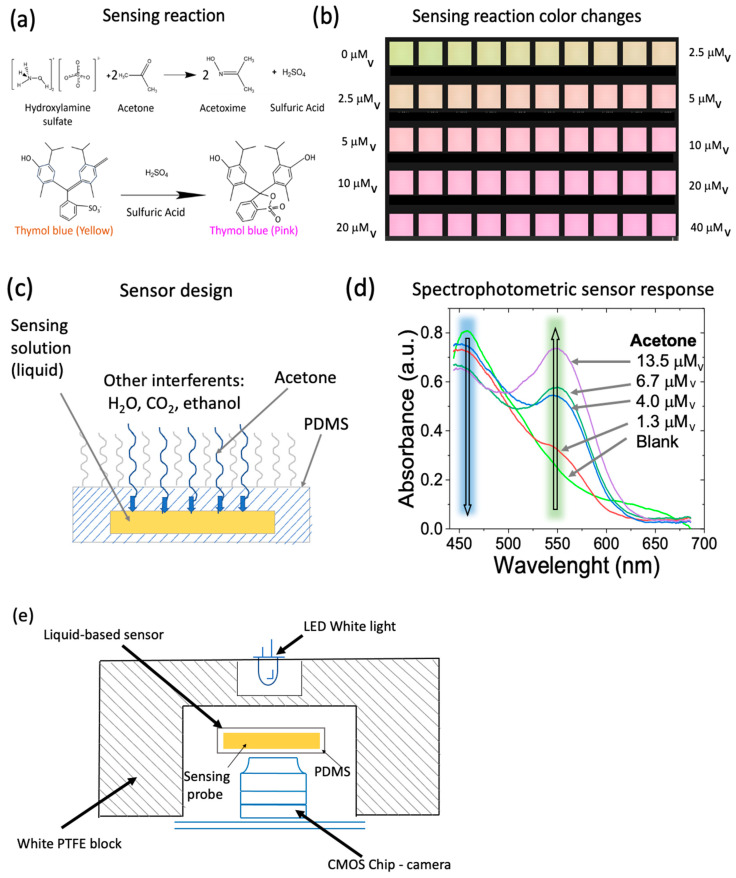
(**a**) Schematic representation of the acetone sensing reaction involving hydroxylamine and thymol blue. (**b**) Sensing reaction color changes upon increasing vapor acetone concentrations. (**c**) Sensor design including hydroxylamine and thymol blue liquid sensing probe and polydimethylsiloxane (PDMS) (Sylgard 184 silicone Elastomer, Midland, MI, USA). (**d**) Spectrophotometric changes in the acetone sensor before and after exposure to different vapor acetone concentrations. (**e**) Configuration of the acetone sensor device. The acetone sensor was located between a white LED and a CMOS chip detector, where the light was transmitted through the sensor.

The acetone detection reaction comprised a single-step reaction of acetone with hydroxylamine acid salt and a colorimetric pH indicator [62]. The reaction of acetone with hydroxylamine results in the release of acid, which causes a local pH change. The pH change is quantified by the color change in the pH indicator, where relatively small amounts of acids cause significant color change (Figure 1a,b). The sensor comprised hydroxylamine sulfate (Acros Organics, Geel, Belgium) and thymol blue (Fluka, Buchs, Switzerland) as pH indicator. This detection method has been extensively applied in the environmental detection of acetone for exposure assessment [63,64]. Other sensors utilizing acetone reaction with hydroxylamine acid salt, but employing different detection methods of the released acid have also been shown on solid substrates [65,66,67]. In this particular work, we explore the analytical performance of the color-based detection reaction in a liquid medium confined to the specific sensor design (see below). 

### 2.2. Sensor Design and Detection Device

The sensor comprising the liquid sensing solution was enclosed in a polymethylsiloxane (PDMS) cavity. Figure 1c describes the sensor design and shows a side view, including the transparent PDMS and the liquid sensing probe in the PDMS internal part. 

Freshly prepared sensors were stored in an aluminized Mylar^®^ bag (Chester, VA, USA), which was sealed in an inert environment with controlled clean air and humidity of ~35%. This allowed to chemically protect the sensors from different humidities, ambient carbon dioxide and other gases, and volatile compounds exposure. The packaged sensors were exposed to different temperature conditions to study the stability of the sensor under storage conditions (see Section 3.4). 

The volume of the liquid sensing solution and thickness of the sensor enclosure were optimized to obtain the acetone sensing maximum sensitivity. We used a UV-visible spectrophotometer (Ocean Optics, Orlando, FL, USA) to determine the spectral changes related to the color change in the sensors (Figure 1c). 

A sensing device was built to study the sensor’s red, blue, and green color response. The device comprised a CMOS chip (Omnivision 5647, Shenzhen ChuangMu Technology, Shenzhen, China) [48] integrated with a white LED source in an optical transmittance mode configuration. The CMOS chip had a fixed-focus module with integral IR filter, a resolution of 5-megapixel 2592 × 1944, a maximum image transfer rate of 1080 p: 30 fps (encode and encode) 72 p: 60 fps, and image control functions of automatic exposure control, white balance, band filter, 50/60 Hz luminance detection, and black level calibration. The dimensions of the CMOS chip were 20 × 25 × 10 mm.

We used a 5 mm white clear LED at the maximum working voltage recommended by the manufacturer of 3 V, which was provided by a DC 9335-PS MPJA power supply. White Teflon light diffuser was placed between the LED and the sensor. For all of the performed tests, the distance between the sensor and the light source was constant.

**Figure 2:**
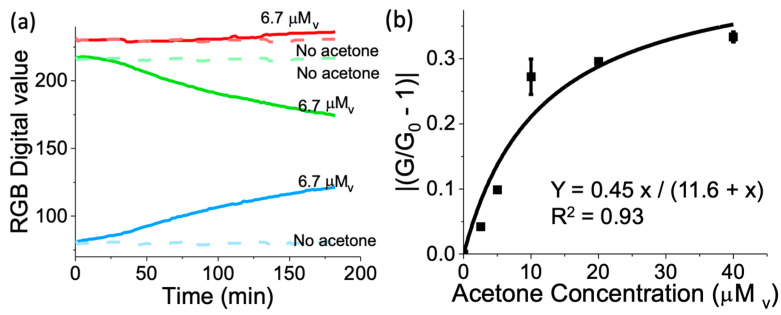

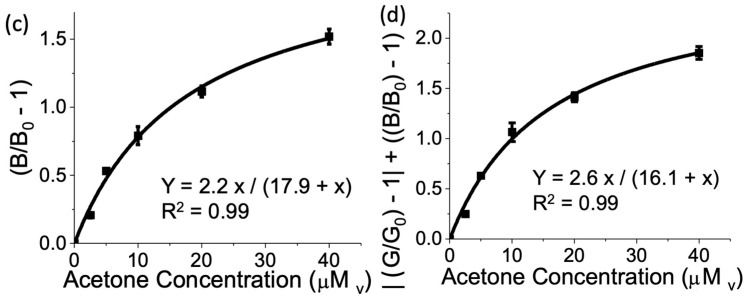
(**a**) RGB components of the sensor response upon exposure to clean air and acetone obtained with a white light emitting diode (LED), complementary metal oxide semiconductor (CMOS) detector, and Matlab^®^ imaging acquisition performing deconvolution of RGB components. (**b**–**d**) Sensor calibration curves: G (**b**), B (**c**), and total (absolute G and B) (**d**) sensor signals as a function of acetone vapor concentration. The sensor response was taken at 100% relative humidity and incubation for 3 h at 32 °C. The experiments were repeated in triplicate for each concentration and had a coefficient of variability < 9.1%.

A polytetrafluorethylene (PTFE) structure was employed to hold the device parts and to act as a light diffuser. The CMOS camera was connected to a Raspberry Pi microcontroller (Raspberry Pi Foundation, Cambridge, UK) to capture the sensor images over time (Figure 1e). Images captured by the CMOS chip were processed by deconvoluting the image color into red (R), green (G), and blue (B) components using a custom algorithm in MatLab^®^ (MathWorks, Natick, MA, USA). The sensor RGB intensity values (I) were recorded in real-time as a function of time (Figure 2a) and processed as follows:

(1) $$\Delta \;Signal=\frac{I}{I_{o}}-1$$

where I represents the intensity of R, G, or B at a given point of the acetone vapor sensing time, and $I_{o}$ represents the intensity of R, G, and B at a time before the reaction. The changes in sensor signal were evaluated as a function of different concentrations of acetone (Figure 2b,d).

Each experiment performed in this work has at least triplicate measurements and the average of the 3 measurements is reported together with its corresponding standard deviation in the error bars. Note that error bars that are not visible have a size smaller than the symbol representing the measurement. More details about sensor and measurement reproducibility are presented in Section 3.2.

### 2.3. Sample Handling

General conditions: To study the sensor performance, we simulated breath samples. This included gas mixtures containing 100% relative humidity. In addition, the sensing was performed at 32 °C, which simulated the breath exhalation temperature by mouth. We prepared artificial samples of acetone with 100% humidity and artificial samples of known concentration with potential interferents at 100% humidity. The interferents included CO_2_ (4%), NH_3_, and ethanol. In addition, we tested CO_2_ interference at different concentrations to further evaluate the interference level in other body fluids such as skin headspace gases. In addition, we used the calibration with CO_2_ for signal processing and breath acetone quantification in real samples. 

Acetone, ammonia, and ethanol testing: Artificial acetone vapor samples were prepared by evaporation of acetone, ammonia, and ethanol solutions of known concentrations in a volume of glass container that was sealed and maintained at 32 °C for a period of 3 h. Volumes between 0.5 and 10 μL of the solutions were introduced into a sensing chamber built from a Glasslock container (Glasslock Inc., Huntington Park, CA, USA). The sensing device was located inside the sensing chamber. It is important to mention that for all interferent testing and breath acetone calibration, we introduced water inside the container to saturate the sensing chamber with water vapor.

In the case of ethanol, a solution of 25 ppb (*v*/*v*) was prepared, 10 µL were placed in the container, and it was tested under the same conditions as the previous ones. For ammonia, a 3.5 ppb (*v*/*v*) solution was prepared, which is the concentration reported for ammonia [47]. For the ammonia solution, ammonium chloride dissolved in a PBS solution at a pH of 7.4 was used. 

Carbon dioxide testing: For interference testing, special attention was taken to test humidity and CO_2_ gas samples, which were prepared in metal laminated Tedlar^®^ bags (Wilmington, Denver, CO, USA) from a certified gas with CO_2_/O_2_ (4%/16% in nitrogen balance) (Carefusion, San Diego, CA, USA). The bags were diluted from the concentrated CO_2_ standard gas, and final concentrations were determined based on the quantification from an IR detector, Carbon Dioxide Analyzer (17630 from Vacumed™, Ventura, CA, USA). Furthermore, the sensing chamber had two 2-way valves to allow for appropriate purging and introduction of the gas stream. With both (input and output) valves opened, the CO_2_ dilutions were injected for 30 s to purge the air inside the sensing chamber. Then, the valves were closed to let the chamber and sensor equilibrate with the injected CO_2_ gas. After this procedure, the sensing chamber was flushed with additional CO_2_ dilution for one minute. Thereafter, the valves were closed to allow the sensing testing to occur under diffusional mass transport conditions with no convection added. 

Breath samples testing: Offline collection of breath samples was performed in 1 L Tedlar^®^ sampling bags with dimensions of 7 in × 7 in. The subject used a non-rebreathing T-valve to breathe into the bag without any flow regulation. A total volume of 1 L was used for each measurement. 

### 2.4. Human Subjects

Informed consent was obtained from all human subjects involved in the research. Human subjects consented to participate under IRB approved protocols at the Arizona State University: STUDY00008255.

#### 2.4.1. Breath Ketone Measurements

The collected breath bag was then transferred to 32 °C and injected into the sensor chamber (made of a Glasslock container (Glasslock USA, Inc.) using two valves to introduce the samples. The sensor chamber was sealed and maintained at 32 °C for a period of 3 h for sensing the breath acetone concentration, reproducing the conditions used to build the calibration curves of the sensor. It is important to notice that during breath collection, a condensation of breath humidity occurs in the Tedlar^®^ bag due to the difference in temperature between the exhaled breath (~32 °C) and the ambient temperature. However, we considered the equilibration of acetone concentration in the gas phase before sensing to avoid the condensation effect.

#### 2.4.2. Standard Blood Ketone Measurements

Blood ketones were measured using Precision Xtra™ electrochemical capillary blood analyzer from Abbott™ (Alameda, CA, USA). The standard procedure described in the analyzer manual with the monitor was employed to test the blood samples. 

## 3. Results

### 3.1. Sensor Calibration

Response of the sensor towards acetone was measured in a range from 0 mM_v_ to 40 mM_v_. The sensor response was assessed as described in the Materials and Methods section for RGB light intensity components (Figure 2a). The highest light intensity changes were observed for green and blue light intensity components. The green light intensity component decreased with the acetone exposure over time, which was indicative of increasing absorbances in the green wavelength range as observed in the spectral changes registered for the sensor upon exposure to acetone vapors in the visible spectrophotometric experiments shown in Figure 1d. On the contrary, the blue light intensity component increased with the acetone exposure over time, which was in correlation with the increasing absorbances in the blue wavelength range observed in the visible spectrophotometric experiments shown in Figure 1d. No significant changes in red light intensity component were observed in coincidence with the visible spectral changes (Figure 1d). 

Figure 2b,d shows the sensor response for the absolute green light intensity changes, blue light intensity changes, and for the additive, absolute green and blue light intensity changes to acetone vapors (evaluated at a fixed time of acetone exposure). In all cases, the sensor response followed a non-linear response with a Langmuir-like behavior. Therefore, we used the Langmuir equation to fit the sensor response, which resulted in a squared-correlation coefficient (R^2^) higher than 0.99. It is worth noticing that we performed three tests at each concentration to build the calibration curves.

### 3.2. Sensor Reproducibility

We tested the sensor responses to assess the reproducibility within a sensor batch and between the sensor batches. We stored the prepared sensors in a clean-air environment prior to testing and tested the response of a fresh sensor upon exposure to 6.7 mM_v_ acetone. The coefficient of variability (CV), defined as the ratio between the sensor response average and sensor response standard deviation, was 4.7% for 40 sensors prepared in the same batch and 19% for sensors prepared between different batches. The within-batch CV was acceptable for analytical standards [68]. We believe the overall good reproducibility of the sensors is due to the streamlined sensor preparation procedure and the sensor signal processing (Equation (1)), which includes $I_{o}$ representing the intensity of R, G, and B at a time before the reaction and eliminates the sensor signal drift that could be caused due any potential adverse condition due to storage.

### 3.3. Sensor Geometry Optimization

**Figure 3:**
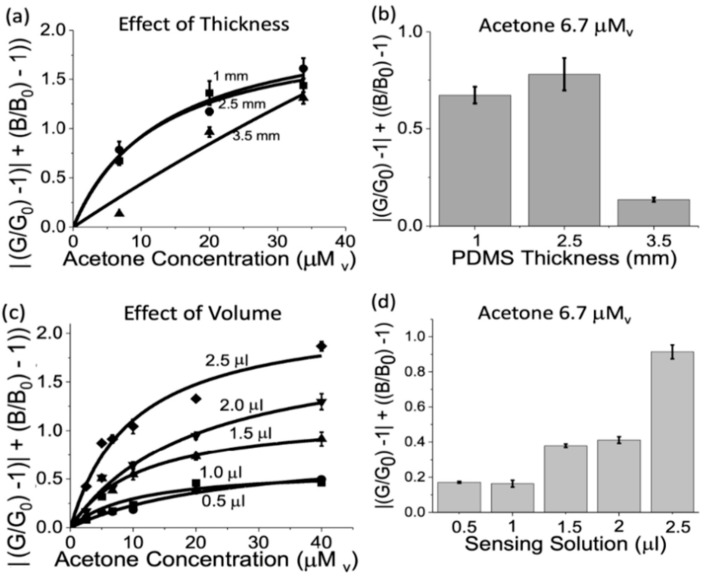
Sensor geometry optimization: Acetone response to different conditions of (**a**,**b**) PDMS thickness for a constant volume of liquid sensing probe (2.5 μL), and (**c**,**d**) liquid sensing probe volume for a constant thickness (2.0 mm). The sensor response was assessed at an acetone vapor concentration from 2.5 to 40 mM_v_ in (**a**,**c**), and of 6.7 mM_v_ in (**b**,**d**), using 100% humidity for 3 h. The total sensor response for green (absolute) and blue light components together with the corresponding Langmuir model fitting are shown in (**a**,**c**).

We observed that the sensitivity of the liquid probe-based acetone sensor can be tuned to different acetone concentration ranges by optimizing the parameters of the sensor: volume of the liquid probe inside the sensor and the thickness of the diffusional barrier (PDMS) between the gas phase and the liquid sensing probe (Figure 1c). Therefore, we systematically investigated these geometrical parameters. Figure 3a,b shows the effect of increasing the PDMS thickness from 1.0 to 3.5 mm, which was controlled by the PDMS weight added to the sensor mold. We observed that the sensor showed an optimal thickness with a maximum response at 2.5 mm. This effect may result from two contributing opposite influential factors: a sensor sensitivity increase with the PDMS thickness increase due to the known PDMS pre-concentrating properties for acetone [69], and a sensor sensitivity decrease with the PDMS thickness increase due to the increase in the diffusional barrier of acetone into the sensing probe. On the other hand, Figure 3c,d shows the effect of increasing the liquid sensing probe volume from 0.5 to 2.5 μL. We observed that increasing the volume inside the sensor increases the sensor response and that a volume of liquid sensing probe of 2.5 μL increases the sensitivity by two-fold with respect to 1.5–2.0 μL and by over four-fold with respect to 0.5–1 μL. This observation was in line with the increasing number of available sensing probe molecules in the sensor.

### 3.4. Sensor Stability

**Figure 4:**
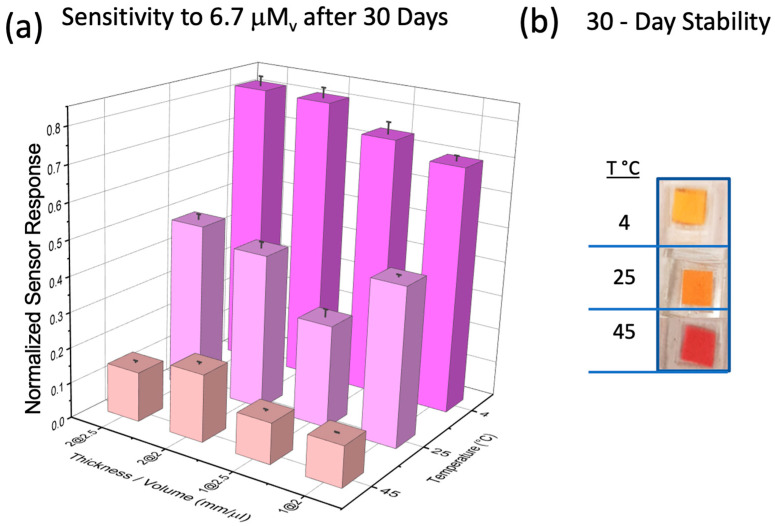
Stability test for acetone sensor over time under different configurations of PDMS thickness, liquid sensing probe volumes, and storage temperature conditions. (**a**) The plot shows the response of the sensors to acetone at a concentration of 6.7 mM_v_ after normalization by the corresponding response assessed on day 1. Each point represents the averaged response for six sensors and the corresponding standard deviation (error bar). The sensor configuration that showed the best stability was the sensor with 2 mm of PDMS thickness and 2.5 μL of liquid sensing probe solution stored at 4 °C for a period of one month. (**b**) Picture of the sensors after exposure to different temperatures for a period of 30 days.

The acetone sensor showed stability towards the sensitive detection of acetone for long periods of time when stored at appropriate temperatures. Figure 4 shows the stability of sensors made of different thicknesses and liquid sensing probe volumes that were stored at 4, 25, and 45 °C. The arrangements were 1 mm and 2 mm thick with 2 and 2.5 microliters, respectively. After one month, the response of the sensors was evaluated and compared with the original values (day 1 of manufacturing). From the data in Figure 4, we can observe that the new liquid sensing probe-based sensor could maintain stability for a month (as tested to date), with the highest stability observed for the sensor with 2 mm of PDMS thickness and 2.5 μL of liquid sensing probe solution stored at 4 °C.

### 3.5. Sensor Interferent Study

**Figure 5:**
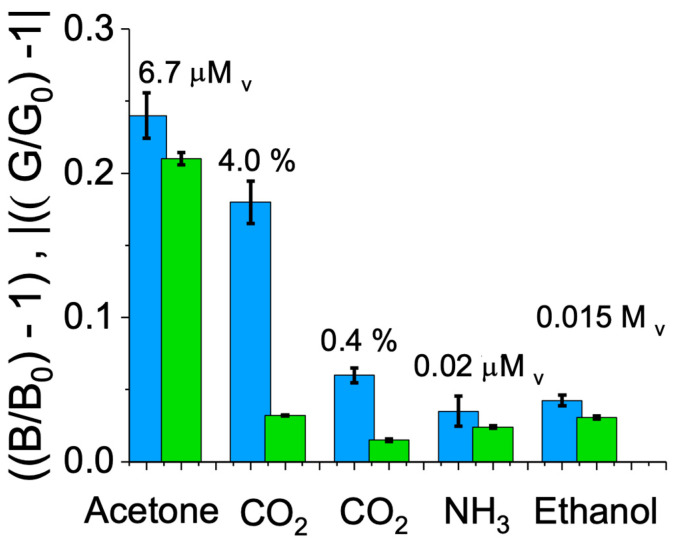
Interferent selectivity analysis of the liquid probe-based sensor comparing detection of acetone versus volatile organic compounds at concentrations found in human samples. Blue and green light intensity sensor signals for acetone, CO_2_, ammonia (NH_3_), and ethanol. The tested ethanol concentration was equivalent to breath ethanol of the “driving-under-influence” threshold condition (0.015 μM_v_ equivalent to 0.0023% *v*/*v*). Tested CO_2_ concentrations were chosen assuming breath (4%) and skin headspace (0.4%) typical concentrations. Tested ammonia concentration was chosen assuming exposure to breath level. Liquid probe-based sensor conditions: 2.0 mm of PDMS thickness, 2.5 μL of sensing probe.

Figure 5 shows the interferent study on the liquid probe-based sensor for potential molecules present in breath and skin. Details of the selection of interferents concentration are presented in the figure captions. From all of the interferents tested, CO_2_ presented an interference response with respect to acetone. We noticed that the cross sensitivity was only present under high humidity conditions, which may be due to the higher diffusion of CO_2_ within the PDMS structure and the formation of carbonic acid, which interacts with the sensing probe’s pH indicator. 

Given that CO_2_ is an interferent of acetone detection in the presence of humidity, we built a system of two calibration equations considering the green and blue light intensity components of the CMOS-based detection method (see the next section).

### 3.6. Sensor Calibration and Real Sample Analysis

**Figure 6:**
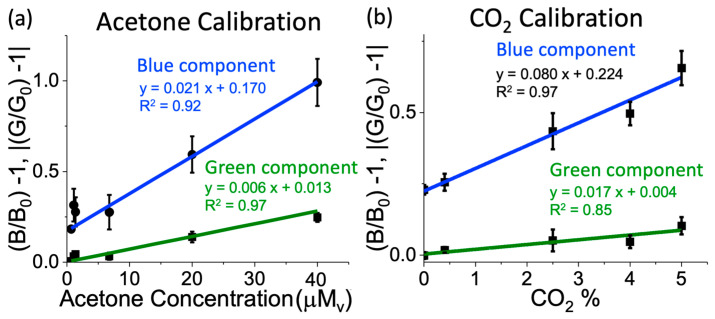
Calibration curves of the liquid probe-based sensor exposed to different concentrations of (**a**) acetone and (**b**) CO_2_. The curves show the calibration equations resulting from the blue and green light intensity components and represent a linear behavior with R^2^ greater than 0.92. Liquid probe-based sensor conditions: 3.5 mm of PDMS thickness, 2.5 μL of sensing probe.

As a consequence of the interferent tests and the cross-sensitivity with carbon dioxide, a sensor calibration procedure was implemented as follows: 1- A series of acetone solutions of known concentration were prepared to generate acetone vapor concentrations between 0 and 40 mMv. 2- Solutions of known concentration of CO_2_ (0–5%) were prepared in the gas phase. 3- The liquid probe-based sensors were exposed to acetone solutions under 100% humidity conditions at 32 °C for three hours, and sensor images were taken before and after the test. 4- A new set of acetone sensors was also exposed to the CO_2_ gas dilutions under the same conditions and time as the previous test with acetone. Figure 6 shows the sensor responses obtained from acetone and CO_2_ with the corresponding fitting equations. Assuming additive properties of the color changes taking place at the same wavelength, we added the equations for acetone and CO_2_ obtained for green and blue light components, respectively, and obtained a system with two equations (green and blue light component) and two unknowns (acetone and CO_2_). The new equation system allowed us to extract the acetone concentration in the presence of CO_2_ under conditions of 100% relative humidity in real samples, such as breath or skin.

It is important to mention that in order to obtain linear sensor responses so that the optical absorption follows an additive property, the sensor’s PDMS thickness was chosen in order that the response of the sensor was linear and diffusively controlled. For this reason, we chose a PDMS thickness of 3.5 mm.

**Figure 7:**
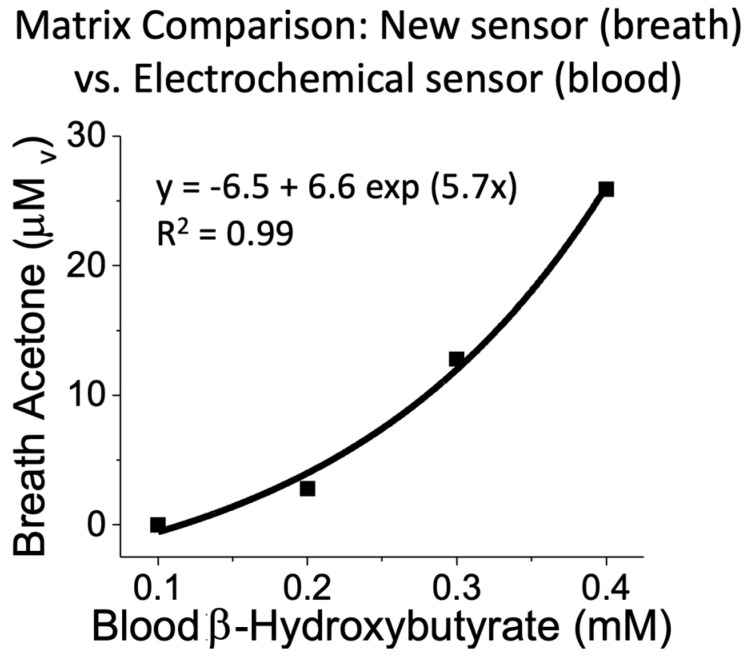
Correlation of field tests between the measurements of the breath acetone levels assessed with the liquid probe-based sensor and the levels of blood β-hydroxybutyrate assessed with Precision Xtra™ electrochemical capillary blood analyzer from Abbott. Liquid probe-based sensor conditions: 3.5 mm of PDMS thickness, 2.5 μl of sensing probe.

To test the accuracy of the sensor, we performed tests on four human subjects that were recruited via ASU IRB (STUDY00008255) and fasted between 10 and 24 h. We collected breath samples from the human subjects in Tedlar^®^ bags, and the breath acetone levels were quantified by the new sensor. In parallel, we analyzed the blood β-hydroxybutyrate levels of the subjects using a reference commercial ketometer: Precision Xtra™ electrochemical capillary blood analyzer from Abbott. Figure 7 shows the correlation between the quantification from our acetone sensor in comparison with the commercial analyzer. Paired *t*-tests of these data showed a *p*-value of 0.28, and therefore, we concluded there were no statistical significances between the breath acetone levels obtained with the new sensor and the blood hydroxybutyrate obtained with the commercial analyzer. In addition, we correlated the acetone levels with b-hydroxybutyrate levels and obtained an exponential relationship with a squared-correlation coefficient (R^2^) from the fitted function equal to 0.99, showing excellent agreement between the measurements. This relationship was similar to previously published values [70,71], which further corroborated the capacity of the liquid probe-based sensor to detect ketone levels and ketone buildup physiological conditions.

## 4. Discussion

In conclusion, a novel liquid-based sensor for measuring acetone has been developed. This sensor can detect breath acetone in a wide concentration dynamic range (0–40 mM_v_). The sensor demonstrated good specificity when used with the blue and green light intensity component calibration equations and relatively good stability when stored at 8 °C. Real breath measurements have shown a strong correlation with the commercial sensor. The sensor device was demonstrated to be useful in monitoring breath acetone due to ketone buildup from fasting. 

The sensor’s successful performance is due to the combination of synergic properties that contribute to the sensor’s robustness, sensitivity, and specificity. These properties include 1- creating liquid probe holding cavities whose volume and dimensions are appropriate to stably hold the liquid over long periods of time (e.g., avoiding evaporation), 2- pre-concentrating the analyte (e.g., acetone) from the body fluid onto areas of polymer in contact with the sensing liquid probe, 3- rejecting volatile organic compounds that could act as interferents of the detection reaction, and 4- providing optimal diffusion thickness to analytes, such as acetone so that acetone can diffuse inside the cavity and react with the sensing probe. Current sensor performance was tested for periods of several minutes to hours. Future sensor improvement will be performed to shorten the sensor time response.

## Data Availability

Data are contained within the article.

## References

[1] Tsow F., Xian X.J., Bridgeman D., Forzani E.S., Tao N.J. (2021). Self-Contained Wearable Metabolic Analyzer.

[2] Forzani E.S., Tao N.J. (2013). Metabolic Analyzer.

[3] Tsow F., Xian X., Forzani E., Tao N.J. (2015). An Improved Portable Metabolic Analyzer System.

[4] Tao N.J., Forzani E.S. (2018). Metabolic Analyzer.

[5] Tsow F., Xian X.J., Forzani E.S., Tao N.J. (2018). Portable Metabolic Analyzer.

[6] Zhao D., Xian X., Terrera M., Krishnan R., Miller D., Bridgeman D., Tao K., Zhang L., Tsow F., Forzani E. (2014). A pocket-sized metabolic analyzer for assessment of resting energy expenditure. J. Clin. Nutr..

[7] Prabhakar A., Quach A., Wang D., Zhang H.J., Terrera M., Jackemeyer D., Xian X., Tsow F., Tao N., Forzani E. (2014). Breath Acetone as Biomarker for Lipid Oxidation and Early Ketone Detection. Glob. J. Obes. Diabetes Metab. Syndr..

[8] Prabhakar A., Quach A., AZhang H., Terrera M., Jackemeyer D., Xian X., Tsow F., Tao N., Forzani E. (2015). Acetone as biomarker for ketosis buildup capability-a study in healthy individuals under combined high fat and starvation diets. Nutr. J..

[9] Wang D., Zhang F., Prabhakar A., Qin X.C., Forzani E.S., Tao N.J. (2021). Colorimetric Sensor for Online Accurate Detection of Breath Acetone. ACS Sens..

[10] McArdle W.D., Katch F.I., Katch V.L. (2010). Exercise Physiology: Nutrition, Energy, and Human Performance.

[11] McGarry J., Foster D. (1980). Regulation of hepatic fatty acid oxidation and ketone body production. Annu. Rev. Biochem..

[12] Hay R., Bond M. (1967). Kinetics of the decarboxylation of acetoacetic acid. Aust. J. Chem..

[13] Reichard Jr G., Owen O., Haff A., Paul P., Bortz W. (1974). Ketone-body production and oxidation in fasting obese humans. J. Clin. Investig..

[14] Atkins R.D. (2002). Dr. Atkins’ New Diet Revolution.

[15] Kossoff E.H., Zupec-Kania B.A., Amark P.E., Ballaban-Gil K.R., Christina Bergqvist A., Blackford R., Buchhalter J.R., Caraballo R.H., Helen Cross J., Dahlin M.G. (2009). Optimal clinical management of children receiving the ketogenic diet: Recommendations of the International Ketogenic Diet Study Group. Epilepsia.

[16] Freeman J.M., Kossoff E.H., Hartman A.L. (2007). The ketogenic diet: One decade later. Pediatrics.

[17] Paoli A., Cenci L., Grimaldi K.A. (2011). Effect of Ketogenic Mediterranean diet with phytoextracts and low carbohydrates/high-protein meals on weight, cardiovascular risk factors, body composition and diet compliance in Italian council employees. Nutr. J..

[18] Kupari M., Lommi J., Ventilä M., Karjalainen U. (1995). Breath acetone in congestive heart failure. Am. J. Cardiol..

[19] Umpierrez G.E., Kitabchi A.E. (2003). Diabetic Ketoacidosis. Treat. Endocrinol..

[20] Hardern R., Quinn N. (2003). Emergency management of diabetic ketoacidosis in adults. Emerg. Med. J. EMJ.

[21] Carroll P., Matz R. (1983). Uncontrolled diabetes mellitus in adults: Experience in treating diabetic ketoacidosis and hyperosmolar nonketotic coma with low-dose insulin and a uniform treatment regimen. Diabetes Care.

[22] Wallace T., Matthews D. (2004). Recent advances in the monitoring and management of diabetic ketoacidosis. Qjm.

[23] Lorenz R.A., Malone J.I., Nathan D.M., Peterson C.M. (2003). Tests of glycemia in diabetes. Diabetes Care.

[24] Csako G. (1987). False-positive results for ketone with the drug mesna and other free-sulfhydryl compounds. Clin. Chem..

[25] Bonnardeaux A., Somerville P., Kaye M. (1994). A study on the reliability of dipstick urinalysis. Clin. Nephrol..

[26] Ham M.R., Okada P., White P.C. (2004). Bedside ketone determination in diabetic children with hyperglycemia and ketosis in the acute care setting. Pediatr. Diabetes.

[27] Cone E.J. (2006). Oral fluid testing: New technology enables drug testing without embarrassment. J. Calif. Dent. Assoc..

[28] Saasa V., Malwela T., Beukes M., Mokgotho M., Liu C.P., Mwakikunga B. (2018). Sensing Technologies for Detection of Acetone in Human Breath for Diabetes Diagnosis and Monitoring. Diagnostics.

[29] Güntner A.T., Kompalla J.F., Landis H., Theodore S.J., Geidl B., Sievi N.A., Kohler M., Pratsinis S.E., Gerber P.A. (2018). Guiding Ketogenic Diet with Breath Acetone Sensors. Sensors.

[30] Musa-Veloso K., Likhodii S.S., Cunnane S.C. (2002). Breath acetone is a reliable indicator of ketosis in adults consuming ketogenic meals. Am. J. Clin. Nutr..

[31] Musa-Veloso K., Likhodii S.S., Rarama E., Benoit S., Liu Y.-M.C., Chartrand D., Curtis R., Carmant L., Lortie A., Comeau F.J. (2006). Breath acetone predicts plasma ketone bodies in children with epilepsy on a ketogenic diet. Nutrition.

[32] Ueta I., Saito Y., Hosoe M., Okamoto M., Ohkita H., Shirai S., Tamura H., Jinno K. (2009). Breath acetone analysis with miniaturized sample preparation device: In-needle preconcentration and subsequent determination by gas chromatography–mass spectroscopy. J. Chromatogr. B.

[33] Tassopoulos C., Barnett D., Russell Fraser T. (1969). Breath-acetone and blood-sugar measurements in diabetes. Lancet.

[34] Musa-Veloso K., Rarama E., Comeau F., Curtis R., Cunnane S. (2002). Epilepsy and the ketogenic diet: Assessment of ketosis in children using breath acetone. Pediatr. Res..

[35] Bovey F., Cros J., Tuzson B., Seyssel K., Schneiter P., Emmenegger L., Tappy L. (2018). Breath acetone as a marker of energy balance: An exploratory study in healthy humans. Nutr. Diabetes.

[36] Španěl P., Smith D. (2011). Progress in SIFT-MS: Breath analysis and other applications. Mass Spectrom. Rev..

[37] Kumar S., Huang J., Abbassi-Ghadi N., Spanel P., Smith D., Hanna G.B. (2013). SIFT-MS Analysis of Exhaled Breath for Volatile Organic Compound Profiling of Esophago-Gastric Cancer. Anal. Chem..

[38] Diskin A.M., Španěl P., Smith D. (2003). Time variation of ammonia, acetone, isoprene and ethanol in breath: A quantitative SIFT-MS study over 30 days. Physiol. Meas..

[39] Turner C., Španěl P., Smith D. (2006). A longitudinal study of ammonia, acetone and propanol in the exhaled breath of 30 subjects using selected ion flow tube mass spectrometry, SIFT-MS. Physiol. Meas..

[40] Smith D., Spanel P., Davies S. (1999). Trace gases in breath of healthy volunteers when fasting and after a protein-calorie meal: A preliminary study. J. Appl. Physiol..

[41] Abbott-Laboratories Abbott Breath Acetone Analyzer. FDA approval pre-market notification: 510 (k). https://www.accessdata.fda.gov/scripts/cdrh/cfdocs/cfpcd/classification.cfm?id=454.

[42] Abbott Breath Acetone Analyzer (1988). FDA 510K application: K875029. https://www.accessdata.fda.gov/scripts/cdrh/cfdocs/cfpmn/pmn.cfm?ID=K875029.

[43] Kundu S.K., George R.W., March S.C., Rutnarak S. (1992). Breath Component Monitoring Device.

[44] Kundu S.K., Bruzek J.A., Nair R., Judilla A.M. (1993). Breath acetone analyzer-Diagnostic tool to monitor dietary fat loss. Clin. Chem..

[45] Landini B.E., Bravard S.T. (2009). Breath Acetone Concentration Measured Using a Palm-Size Enzymatic Sensor System. IEEE Sens. J..

[46] Yamada Y., Hiyama S. (2013). Breath Acetone Analyzer to Achieve “Biochip Mobile Terminal”. NTT DOCOMO Tech. J..

[47] Forzani E. (2016). Acetone Breath Analysis system: Reusable, no continuous monitoring, semiconductor technology with limited selectivity (high interferents’ level)..

[48] Alkedeh O., Priefer R. (2021). The Ketogenic Diet: Breath Acetone Sensing Technology. Biosensors.

[49] Ahmad L.M., Satterfield B.C., Martineau R.L. (2019). Method and Apparatus for Analyzing Acetone in Breath.

[50] Li J., Smeeton T.M., Zanola M., Barrett J., Berryman-Bousquet V. (2018). A compact breath acetone analyser based on an ultraviolet light emitting diode. Sens. Actuators B Chem..

[51] Yu J.-B., Byun H.-G., So M.-S., Huh J.-S. (2005). Analysis of diabetic patient’s breath with conducting polymer sensor array. Sens. Actuators B Chem..

[52] Xiong X., Xia M. (2012). Carbon Nanotube-Based Ultra-Sensitive Breath Acetone Sensor for Non-Invasive Diabetes Diagnosis. https://core.ac.uk/reader/52955880.

[53] Righettoni M., Tricoli A., Gass S., Schmid A., Amann A., Pratsinis S.E. (2012). Breath acetone monitoring by portable Si: WO_3_ gas sensors. Anal. Chim. Acta.

[54] Neri G., Bonavita A., Micali G., Donato N. (2010). Design and development of a breath acetone MOS sensor for ketogenic diets control. Sens. J. IEEE.

[55] Ahmad L.M., Ahmad S.A., Smith Z. (2019). Breath Ketone Measurement System with Analysis Unit that Communicates with Mobile Application.

[56] Ahmad L.M., Satterfield B.C., Martineau R.L. (2019). Method and Apparatus for Analysing Acetone in Breath.

[57] Ahmad L.M., Satterfield B.C., Martineau R.L. (2020). Method and Apparatus for Analyzing Acetone in Breath.

[58] Ahmad L.M., Satterfield B.C., Martineau R.L. (2017). Method and Apparatus for Analyzing Acetone in Breath.

[59] Ahmad L.M., Satterfield B., Martineau R. (2015). Method and Apparatus for Analyzing Acetone in Breath.

[60] Ahmad L.M., Satterfield B., Martineau R. (2016). Method and Apparatus for Analyzing Acetone in Breath.

[61] Ahmad L.M., Smith Z.B., Ahmad S.A., Kim C. (2020). Measuring an Analyte in Breath Using a Porous Structure Containing a Reactant.

[62] Marasco M. (1926). Hydroxylamine Hydrochloride for the Quick Estimation of Acetone. Ind. Eng. Chem..

[63] Nakano N., Nagashima K. (1999). An automatic monitor of formaldehyde in air by a monitoring tape method. J. Environ. Monit..

[64] http://www.zefon.com/analytical/download/151d.pdf.

[65] Massick S.M., Vakhtin A. Breath acetone detection. Proceedings of the Optics East 2006.

[66] Deng C., Zhang J., Yu X., Zhang W., Zhang X. (2004). Determination of acetone in human breath by gas chromatography-mass spectrometry and solid-phase microextraction with on-fiber derivatization. J. Chromatogr. B.

[67] Tsai S.-W., Que Hee S.S. (2000). A new passive sampler for regulated workplace ketones. AIHAJ-Am. Ind. Hyg. Assoc..

[68] Kaplan A.K., Pesce A.J.E. (1989). Clinical Chemistry: Theory, Analysis, Correlation.

[69] Grote C., Pawliszyn J. (1997). Solid-Phase Microextraction for the Analysis of Human Breath. Anal. Chem..

[70] Rooth G., Carlström S. (1970). Therapeutic fasting. Acta Med. Scand..

[71] Reichard G.A., Haff A.C., Skutches C.L., Paul P., Holroyde C.P., Owen O.E. (1979). Plasma acetone metabolism in the fasting human. J. Clin. Investig..

